# MCM9 deficiency impairs DNA damage repair during spermatogenesis, leading to Sertoli cell-only syndrome in humans

**DOI:** 10.1038/s41420-025-02581-y

**Published:** 2025-07-01

**Authors:** Xuan Sha, Xin Zhang, Hao Geng, Yuqian Li, Xun Xia, Guotong Li, Rong Hua, Kuokuo Li, Yang Gao, Qunshan Shen, Rui Guo, Yuping Xu, Xiaojin He, Yunxia Cao, Mingxi Liu, Huan Wu

**Affiliations:** 1 Reproductive Medicine Center, Department of Obstetrics and Gynecology, the First Affiliated Hospital of Anhui Medical University, Hefei, 230022 China; 2 State Key Laboratory of Reproductive Medicine and Offspring Health, The Affiliated Taizhou People’s Hospital of Nanjing Medical University, Taizhou School of Clinical Medicine, Nanjing Medical University, Nanjing, 211166 China; 3 NHC Key Laboratory of Study on Abnormal Gametes and Reproductive Tract (Anhui Medical University), Hefei, 230032 China; 4 Key Laboratory of Population Health Across Life Cycle (Anhui Medical University), Ministry of Education of the People’s Republic of China, Hefei, 230032 China; 5 Anhui Province Key Laboratory of Reproductive Disorders and Obstetrics and Gynaecology Diseases, Hefei, 230032 China; 6 Biopreservation and Artificial Organs, Anhui Provincial Engineering Research Center, Anhui Medical University, Hefei, 230032 China; 7 Reproductive Medicine Center, Department of Obstetrics and Gynecology, Shanghai General Hospital, Shanghai Jiao Tong University School of Medicine, No 650 Xinsongjiang Road, Shanghai, 200080 China; 8 Center for Big Data and Population Health of IHM, Anhui Medical University, Hefei, 230032 China

**Keywords:** Infertility, Disease genetics

## Abstract

Non-obstructive azoospermia (NOA) represents the most severe form of male infertility; however, its genetic etiology remains largely elusive. MCM9 is crucial for DNA damage repair in mammalian somatic cells, playing a key role in regulating both homologous recombination (HR) and mismatch repair (MMR) pathways. In mice, MCM9 deficiency leads to spermatogenic failure characterized by progressive germ cell depletion and impaired HR repair. However, the underlying mechanism remains unclear in humans. Our study identified two novel homozygous loss-of-function (LoF) mutations in MCM9 in two unrelated NOA patients presenting with Sertoli cell-only syndrome (SCOS). The absence of testicular MCM9 confirmed the pathogenicity of these LoF mutations. Furthermore, diminished HR-mediated DNA repair capacity observed in HEK293T cells, either lacking MCM9 or overexpressing mutant MCM9 plasmids, highlighted the deleterious impact of these LoF mutations on HR repair. Additionally, the confirmed interaction between human testicular MCM9 and both MSH2 and MLH1, alongside findings that human MCM9 is predominantly expressed in spermatogonial stem cells and spermatogonia, provides compelling evidence for the involvement of the MCM9-mediated MMR pathway in maintaining genomic integrity and supporting the viability and proliferation of spermatogonia in humans. Given the poor outcomes of microdissection testicular sperm extraction (micro-TESE) observed in both probands, we propose that biallelic LoF mutations in MCM9 may serve as non-invasive molecular biomarkers for predicting micro-TESE failure. These findings enhance our understanding of the genetic basis of human NOA, particularly SCOS, and provide valuable insights for genetic counseling and fertility guidance tailored to these patients.

## Introduction

Mammalian spermatogenesis is a complex, multi-step process that is critical for male fertility. This process encompasses three pivotal stages: spermatogonial phase, which involves the proliferation and differentiation of spermatogonia into diploid primary spermatocytes; meiotic division, in which haploid spermatids are formed from diploid spermatocytes; and spermiogenesis, during which round spermatids undergo extensive biochemical and morphological transformations. These changes include chromatin remodelling, acrosome formation, and flagellum assembly, culminating in the differentiation of mature spermatozoa [1]. Defects at any of these stages are frequently linked to severe spermatogenic failure (SPGF), which clinically manifests as non-obstructive azoospermia (NOA), a condition characterised by the complete absence of sperm in the ejaculate [2]. This devastating condition represents the most serious form of male infertility, affecting approximately 1% of reproductive-aged men worldwide [3]. The aetiology of NOA remains a key research focus in human reproduction. Chromosomal abnormalities and Y-chromosome microdeletions contribute to a fraction of cases, and monogenic defects are increasingly being recognized as the primary factors in this genetically diverse condition [4]. Consistent with this view, more than 1000 genes specifically participate in mammalian spermatogenesis, and mutations in more than 400 genes cause SPGF in animal models [5, 6]. Despite these insights, current research has elucidated only a limited subset of genetic anomalies associated with human NOA [7, 8]. Notably, over 70% of idiopathic NOA cases remain etiologically undefined [9], suggesting that additional genetic factors may underlie the extensive number of unexplained NOA cases.

Species propagation, a foundational tenet of biology, encapsulates the core essence of life. For patients with NOA, achieving biological parenthood is primarily facilitated by advanced reproductive technologies. Microdissection testicular sperm extraction (micro-TESE) coupled with intracytoplasmic sperm injection remains the most advanced and effective strategy [10]. However, challenges such as low sperm retrieval rate (SRR), risk of iatrogenic testicular trauma, and significant psychological impact associated with this invasive procedure highlight the urgent need for non-invasive molecular biomarkers that can predict preoperative micro-TESE outcomes. Notably, the SRR in patients with idiopathic NOA is significantly lower than that in those with Klinefelter syndrome or AZFc microdeletions [11], underscoring the need for genetic predictors to perdict sperm retrieval outcomes in idiopathic NOA cases prior to surgery [12]. In this context, biallelic mutations in meiosis-associated genes are associated with poor micro-TESE outcomes, whereas genetic defects that disrupt spermatogenesis post-meiosis typically correlate with favourable SRRs [12, 13]. Thus, genetic diagnosis of idiopathic NOA is of great significance, particularly given its prognostic value in predicting the success of sperm retrieval.

Minichromosome Maintenance 9 Homologous Recombination Repair Factor (*MCM9*, OMIM: 610098) encodes a member of the MCM protein family that is crucial in initiating DNA replication in all eukaryotic cells [14]. Predominantly expressed in mammalian gonads, MCM9 plays a vital role in gametogenesis by supporting homologous recombination (HR) repair and maintaining genomic integrity during cell division. In mice, MCM9 deficiency results in gametogenic defects, characterised by germ cell depletion due to impaired HR-mediated DNA repair [15, 16]. Clinically, *MCM9* mutations in humans are predominantly associated with primary ovarian insufficiency (POI) in females [17–19]. To date, only a few biallelic mutations linked to NOA have been briefly reported [20–22]. However, comprehensive functional studies elucidating the precise biological role of MCM9 in human spermatogenesis, as well as detailed evaluations of micro-TESE outcomes in NOA cases with MCM9 mutations, are still lacking.

Herein, we identified two novel homozygous mutations in *MCM9*–a splicing mutation and a nonsense mutation–in two unrelated Chinese patients with NOA. The pathogenicity of these mutations was evaluated by analysing the expression levels of testicular MCM9 in both probands and assessing the HR-mediated DNA repair capacity in HEK293T cells lacking MCM9 or overexpressing mutant MCM9 plasmids. Additional investigations were conducted to examine the association between MCM9 and the DNA mismatch repair (MMR) pathway during human spermatogenesis and to evaluate the outcomes of micro-TESE in these patients. These findings highlight the importance of MCM9 in human spermatogenesis and provide valuable information regarding genetic counselling and fertility guidance for patients harboring *MCM9* mutations.

## Results

### Identification of novel homozygous LoF mutations in *MCM9* as potential causative factors for human NOA

Preliminary filtered WES data were further analysed following the process described in our previous studies [23, 24]. The consanguineous origin of both probands indicated a possible recessive inheritance pattern for NOA; therefore we prioritised the investigation of genes with rare homozygous, hemizygous, or complex heterozygous mutations. Special emphasis was placed on the genes expressed in the testes or those potentially implicated in mammalian spermatogenesis. Through this tiered filtering strategy, we first identified mutations with minor allele frequencies <1% in population databases (gnomAD, ExAC, and 1000 Genomes) and assessed pathogenicity by consensus algorithms (Mutation Taster, SIFT, PolyPhen-2, and CADD; Supplemental Table 2 and 3). Subsequent functional filtering excluded genes lacking spermatogenic involvement (e.g., *SLAIN1*, *AGAP3*, *LMBRD1*, *GALNT12*, *PRICKLE3*, *ABHD17A*). While *SLC9B1*—a testis-specific sodium-hydrogen exchanger—was previously linked to defective sperm motility via impaired sAC-cAMP signaling [25], it showed no association with SPGF in our cohort. Finally, a homozygous splicing mutation in *MCM9* (c.1151-1 G > A) was identified as a pathogenic factor in both the NOA-affected brother (AN013, family member II-1) and his POI-affected sister (family member II-3). Additionally, a homozygous nonsense mutation in *MCM9* (c.1891C>T, p.Gln631X) was discovered in the proband AN020 after excluding candidate genes without established involvement in spermatogenesis (Supplemental Table 1 and 4).

**Fig. 1:**
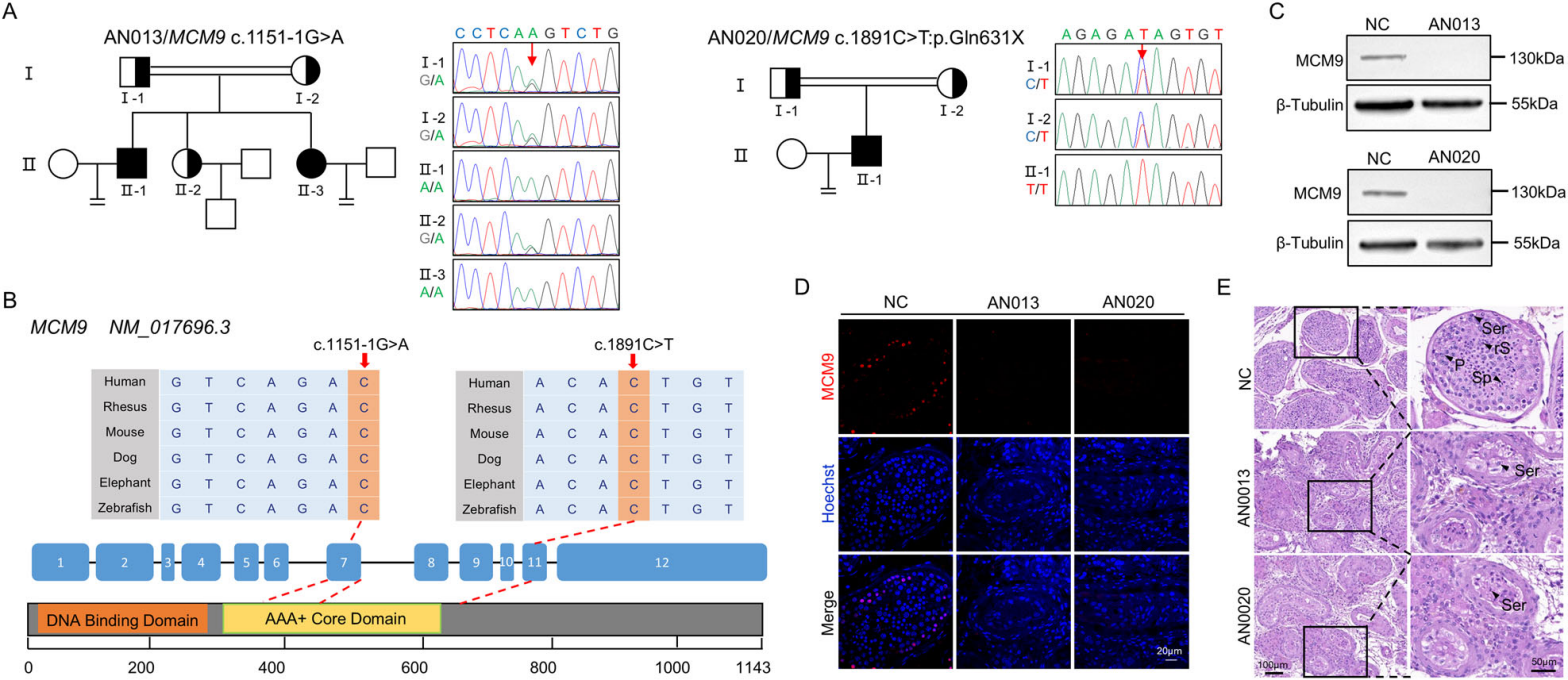
Homozygous loss-of-function mutations in *MCM9* were identified in two unrelated patients with non-obstructive azoospermia. **A** Pedigree analysis and Sanger sequencing of two affected consanguineous families. **B** Evolutionary conservation and exon location of mutations *MCM9*^1151-1G>A^ and *MCM9*^1891C>T^. **C** Western blotting (WB) analyses of MCM9 expression in testicular samples from the two probands and a control male, with β-Tubulin as the loading control. **D** Immunofluorescence (IF) staining of the testis sections with anti-MCM9 antibody (red) and Hoechst (blue) for the two probands and a control male. Scale bar, 20 μm. **E** Hematoxylin and eosin staining of testis sections from the two probands and a control male. Enlarged images show seminiferous tubules with only Sertoli cells in the mutant samples. Scale bar, 50 and 100 μm. NC, normal control; Ser, Sertoli cell; P, pachytene spermatocyte; rS, round spermatid; SP, spermatid.

Sanger sequencing validated the presence of these mutations and showed that they originated from heterozygous, asymptomatic parents (Fig. 1A). Bioinformatic predictions classified these mutations as highly deleterious (Table 1), and the mutant residues showed evolutionary conservation across various mammalian species, including both primates and non-primates (Fig. 1B). To evaluate the pathogenic effects of these novel mutations further, we conducted WB and IF analyses of testicular samples from both probands, with a control male sample included for comparison. The results revealed the complete absence of the MCM9 protein in the mutant testes, with no truncated forms detected (Fig. 1C and Supplemental Fig. 1), and no MCM9-immunostaining foci were observed within the mutated seminiferous tubules (Fig. 1D).

**Table 1:** Clinical evaluation of NOA patients carrying biallelic mutations in *MCM9*.

	Patients	
	AN013	AN020
Genetics analysis		
cDNA mutation^a^	c.1151-1 G > A	c.1891C>T
Protein alteration^b^	NA	p.Gln631X
Exon	Exon7	Exon11
Mutation type	Splicing	Nonsense
Allele frequency		
1KGP	NA	NA
ExAC	NA	NA
gnomAD	0	NA
Deleterious prediction		
Mutation Taster	D	D
CADD	28.6	42
**Clinical characteristics**		
Age (years)	32	34
Semen parameters		
Semen volume (mL)	3.5	2.0
Semen PH	7.2	7.2
Sperm concentration (10^6^/mL)	0	0
Testicular volume (mL)		
Right side	5.0	8.0
Left side	5.0	5.0
Sex hormone levels		
FSH (mIU/mL)	18.81	24.05
LH (mIU/mL)	3.16	13.25
T (nmol/L)	8.65	9.45
E2 (pmol/L)	55.0	18.35
PRL (ng/mL)	7.93	15.50
Karyotype	46, XY	46, XY
AZF deletion	Undetectable	Undetectable
**Sperm retrieval outcomes**		
micro-TESE	No	No

a, The GenBank accession numbers of MCM9 is NM_001029860; b, Full length protein has 708 amino acids.

Abbreviations: NOA, non-obstructive azoospermia; 1KGP, 1000 Genomes Project; ExAC, Exome Aggregation Consortium; gnomAD, Genome Aggregation Database; NA, not available; D, disease causing; FSH, follicle stimulating hormone; LH, luteinizing hormone; T, testosterone; E2, estradiol; PRL, prolactin; AZF, azoospermia factor; micro-TESE, microdissection testicular sperm extraction.

### c.1151-1 G > A mutation causes skipping of exon 7, resulting in truncated and unstable transcript of *MCM9*

**Fig. 2:**
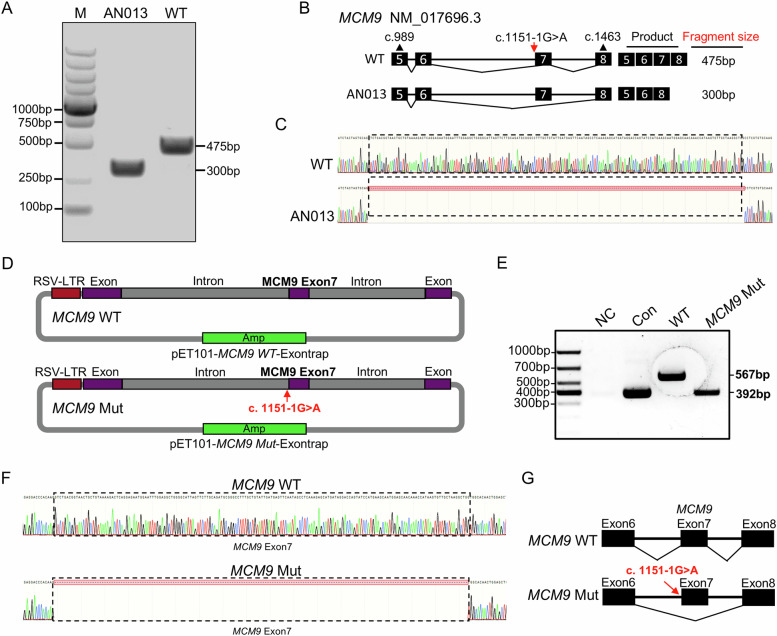
Functional analysis of the *MCM9*^c.1151-1G>A^ mutation to assess its impact on pre-mRNA splicing. **A–C** PCR amplification and cDNA sequence of the *MCM9* cDNA spanning exon 5 to 8 using testicular samples from the two probands and a control male. **D**–**G** Minigene assays showing abnormal pre-mRNA splicing of *MCM9* induced by the c.1151-1 G > A mutation in vitro. NC, normal control; Mut, mutant; WT, wild type.

To evaluate the impact of the novel c.1151-1 G > A mutation on pre-mRNA splicing, *MCM9* cDNA from the testicular cells of AN013 and a male control were analysed. Analysis of the regions encompassing exons 5 and 8 of *MCM9* revealed that the control sample had an intact *MCM9* transcript of the expected length of 475 base pairs (bps), whereas the proband had a truncated *MCM9* transcript of only 300 bp (Fig. 2A). Sanger sequencing of this 300-bp transcript revealed the absence of the entire 175 bp of exon 7, indicating that this splicing mutation led to exon 7 skipping during *MCM9* transcription in vivo (Fig. 2B, C). The absence of the MCM9 protein in mutant testes suggested that this truncated transcript was unstable and likely subject to nonsense-mediated mRNA decay. Further exon-trapping/minigene assays conducted in vitro validated the harmful effects of this mutation at the mRNA level (Fig. 2D). RT-PCR detected a 392-bp band in the mutant condition, whereas a longer 576-bp band corresponding to a properly spliced product was identified in the WT condition (Fig. 2E). Sanger sequencing confirmed that exon 7 skipping was induced by a splicing mutation, which was consistent with our in vivo findings (Fig. 2F and G).

### MCM9 deficiency causes complete depletion of germ cells, leading to SCOS in humans

**Fig. 3:**
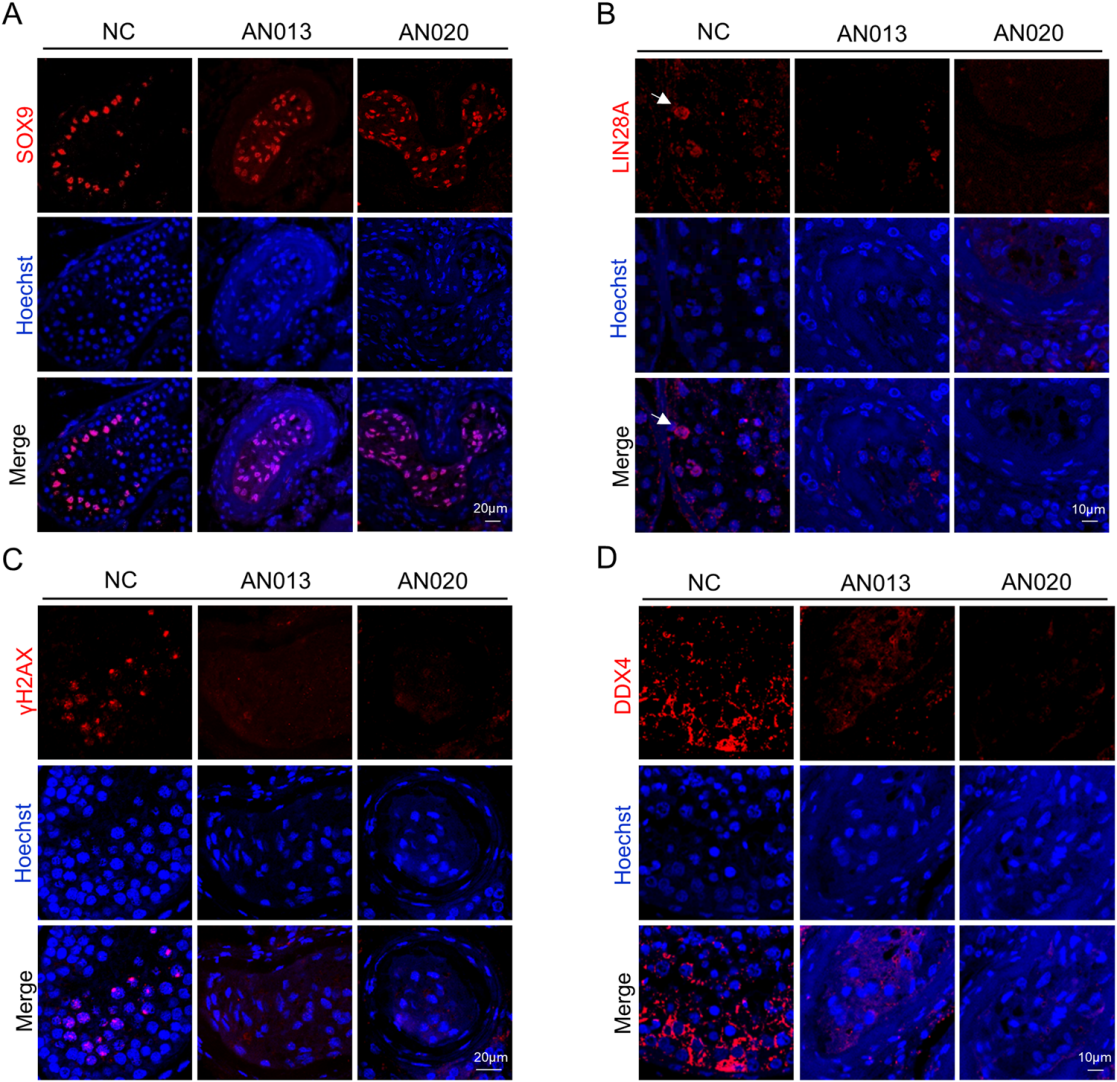
Identification of Sertoli cell-only syndrome in both probands. **A**–**D** IF staining of testis sections with various markers: anti-SOX9 antibody (red), anti-LIN28A antibody (red), anti-γH2A.X antibody (red), anti-DDX4 antibody (red), and Hoechst (blue) for the two probands and a control male. Scale bar, 10 and 20 μm. NC, normal control.

The clinical characteristics of the two familial cases of NOA (AN013) and POI and the individual patient with NOA (AN020) are detailed in Table 1 and Supplemental Table 5, respectively. Both probands presented with reduced testicular volume and elevated serum follicle-stimulating hormone, consistent with classic NOA indicators [26]. Histopathological analysis revealed thickened basement membranes and significant luminal narrowing with vacuolisation in mutant seminiferous tubules. Furthermore, these seminiferous tubules appeared to contain only Sertoli cells, with a complete absence of germ cells, in contrast to the normal spermatogenesis observed in the control male (Fig. 1E). IF staining was conducted to confirm these findings, utilising markers specific to undifferentiated spermatogonia (LIN28A), spermatocytes (γH2AX/DDX4), and Sertoli cells (SOX9). The results demonstrated the exclusive presence of SOX9 signals within the lumen of mutant seminiferous tubules (Fig. 3A), with no signal detected for LIN28A, γH2AX, and DDX4 (Fig. 3B–D). This observation further confirmed the complete depletion of germ cells, including spermatogonia and spermatocytes, in the mutant testes, highlighting the strong association between MCM9 deficiency and SCOS in humans.

### Human testicular MCM9 is predominately expressed in spermatogonia and spermatocytes

**Fig. 4:**
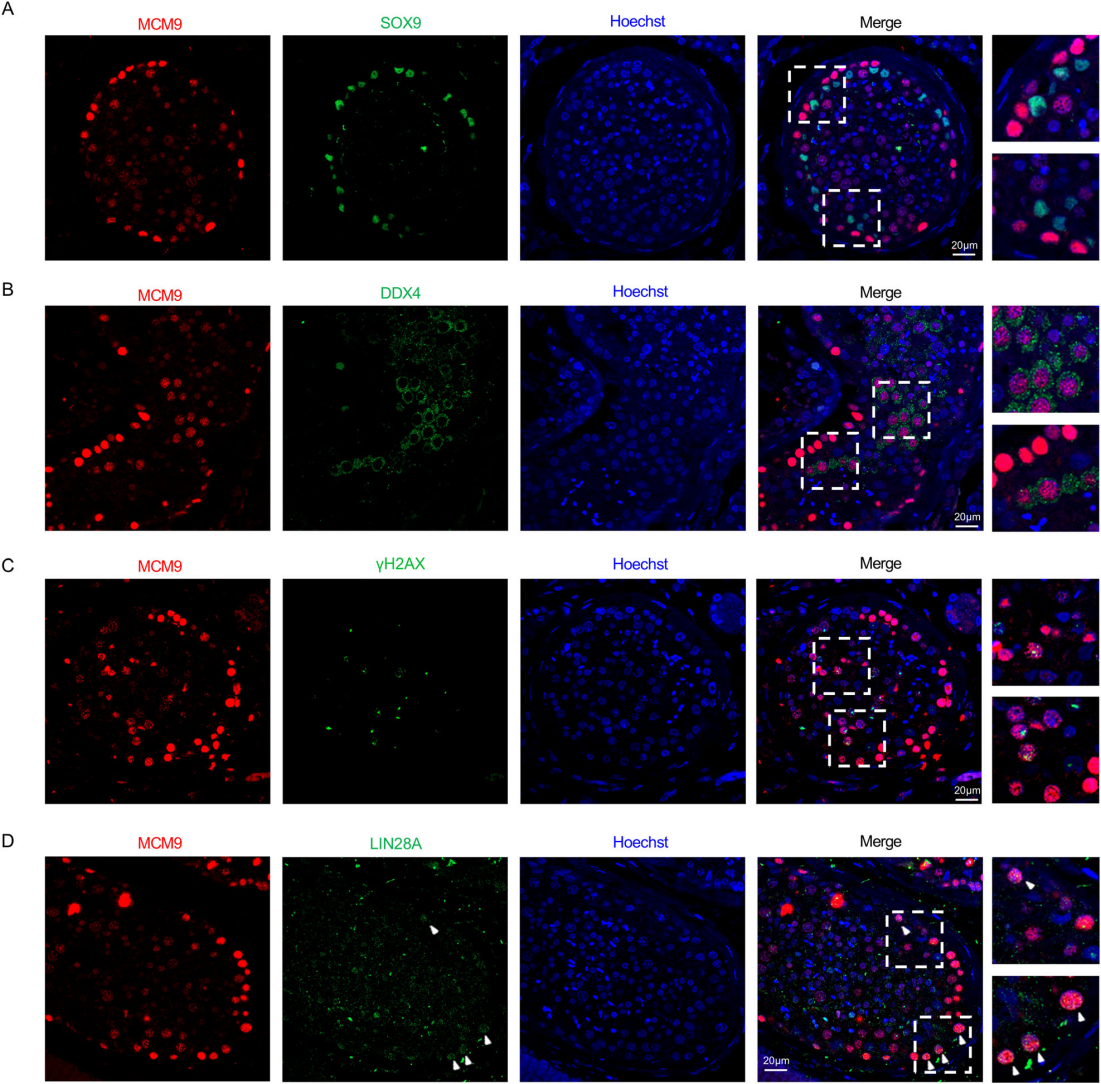
Expression profiles of MCM9 in human testicular cells. **A**–**D** IF staining of human testis sections using specific markers: anti-SOX9 antibody (green), anti-DDX4 antibody (green), anti-γH2A.X antibody (green), anti-LIN28A antibody (green), anti-MCM9 antibody (red), and Hoechst (blue). Scale bar, 20 μm.

To characterize the distinct expression patterns of MCM9 in adult testicular cells of humans and mice, we performed immunofluorescence staining to examine the localization of MCM9 in human testicular tissue. Our findings revealed colocalization of MCM9 with DDX4, γH2AX, and LIN28A, suggesting that *MCM9* is predominantly expressed in spermatogonia and spermatocytes (Fig. 4A–D). In addition, analysis of publicly available scRNA-seq data from testicular tissue demonstrated that *MCM9* mRNA is highly expressed in spermatogonial stem cells (SSCs), spermatogonia, and spermatocytes in both human and mouse testes (Supplemental Fig. 2A, B). The specific subcellular localisation of MCM9 in the mammalian testes highlights its critical role in spermatogonial viability and proliferation. This observation is particularly significant, as it correlates with the severe germ cell depletion phenotype in the *Mcm9*^KO^ mouse model [16], providing further insights into the pathogenesis of SCOS in humans associated with MCM9 deficiency.

### MCM9 deficiency impairs DNA damage repair capacity in vitro

**Fig. 5:**
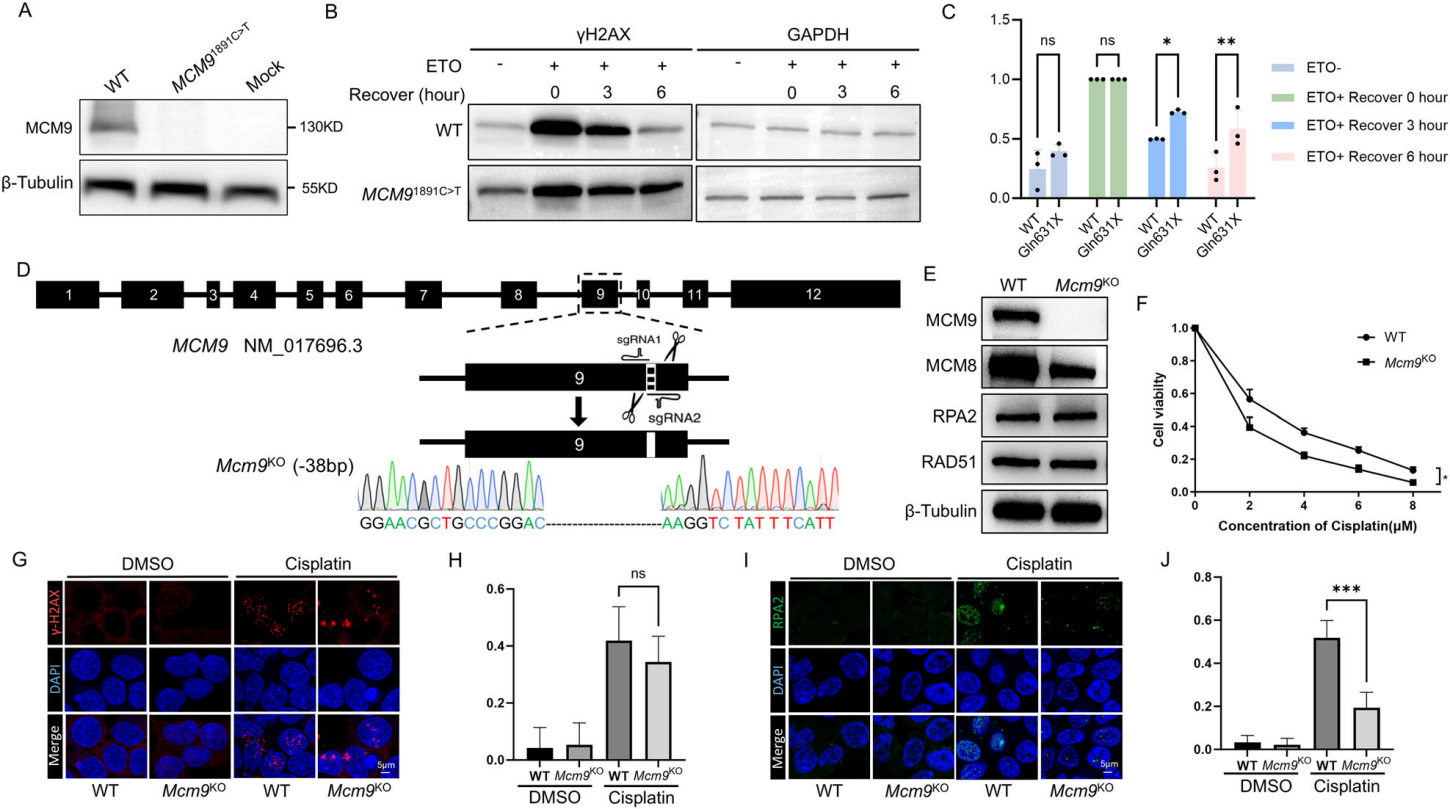
MCM9 deficiency impairs the capacity for DNA damage repair in vitro. **A**–**C** DNA repair assays conducted on HEK293T cells overexpressing the MCM9 mutant (c.1891C>T). **D** Overview of the CRISPR/Cas9 knockout strategy for targeted gene editing in HEK293T cells. **E** WB analysis of MCM9, MCM8, RPA2, and RAD51 expression levels in Mcm9 knockout (*Mcm9*^KO^) HEK293T cells, with β-Tubulin used as a loading control. (**F**–**J**) DNA repair assays in *Mcm9*^KO^ HEK293T cells.

To evaluate the effect of MCM9 deficiency on DNA repair efficacy, we co-transfected HEK293T cells with either mutant or WT MCM9 plasmids and exposed them to ETO, a potent inducer of DNA interstrand crosslinking (ICLs). We assessed the DNA repair efficiency by quantifying γH2AX levels, a marker of DSBs. In cells overexpressing WT MCM9, γH2AX levels increased sharply after ETO treatment and subsequently decreased during a 6-h recovery period. Conversely, cells overexpressing the mutant c.1891C>T that disrupts MCM9 synthesis (Fig. 5A), maintained significantly elevated γH2AX levels at 3 and 6 h post-recovery compared to those in WT cells (Fig. 5B and C). These results indicated that MCM9 deficiency reduced the efficiency of DSB repair, leading to persistent DNA damage.

Given that MCM9 was expressed in germ and somatic cells, we generated *Mcm9*^KO^ HEK293T cells (Fig. 5D) and exposed them to other sources of DSB to evaluate the effects of MCM9 deficiency on DNA damage repair. WB blotting confirmed the absence of MCM9 in *Mcm9*^KO^ cells, with reduced MCM8 expression and unchanged levels of PRA2 and RAD51 (Fig. 5E). This observation wss consistent with the conclusion that MCM8 and MCM9 form a complex to facilitate HR during DNA repair [27]. Subsequently, the WT and *Mcm9*^KO^ cells were treated with escalating doses of cisplatin, another ICL-inducing agent. With increasing cisplatin concentrations, the viability of both cell populations progressively declined. Notably, the viability of *Mcm9*^KO^ cells was significantly lower than that of WT cells, suggesting compromised repair capability of *Mcm9*^KO^ cells in response to cisplatin-induced toxicity (Fig. 5F). IF staining showed that, similar to those under ETO treatment, γH2AX signals were prominent in both WT and *Mcm9*^KO^ cells upon exposure to cisplatin, with no significant difference in the proportion of cells exhibiting γH2AX signals between the two cell types (Fig. 5G, H). However, over the same duration, the number of cells displaying RPA2 signals in *Mcm9*^KO^ cells was notably lower than that in WT cells (Fig. 5I, J). This suggests that *Mcm9*^KO^ cells have defects in PRA2 recruitment, indicating a considerable decrease in DSB their repair capability in *Mcm9*^KO^ cells.

### Deficient HR and MMR may underlie impairment of DNA damage repair caused by MCM9 deficiency

**Fig. 6:**
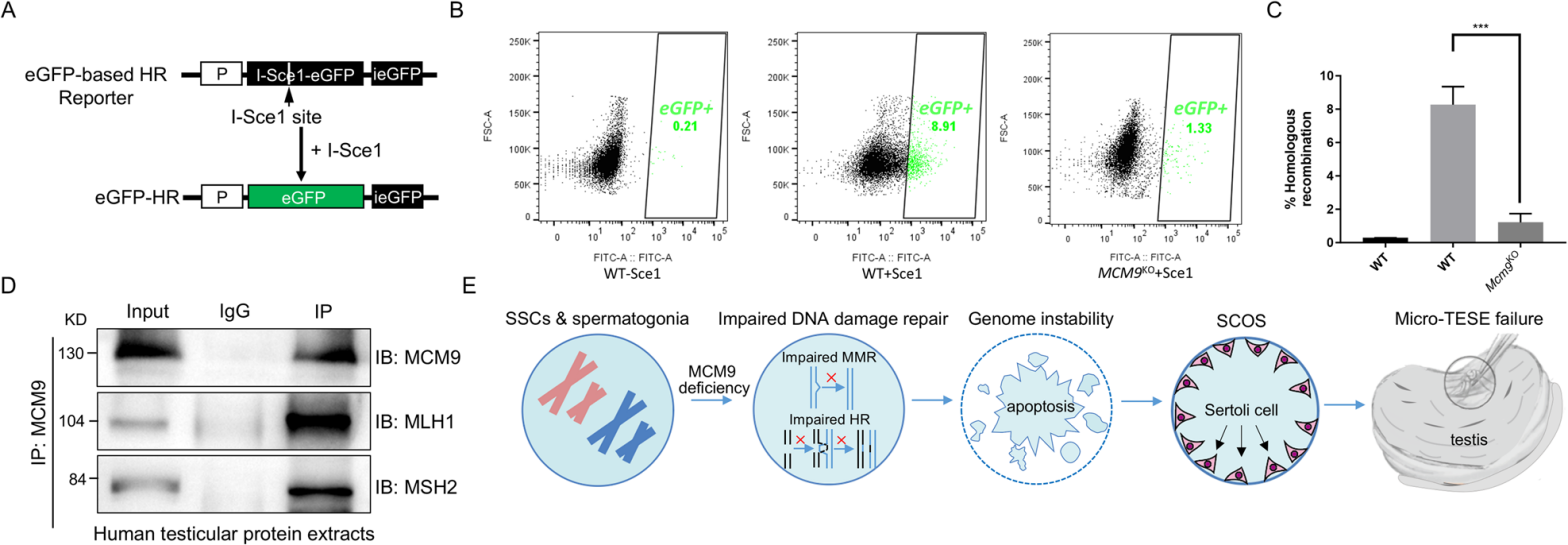
Deficient homologous recombination (HR) and mismatch repair may underlie the impaired DNA damage repair caused by MCM9 deficiency, ultimately leading to the failure of microdissection testicular sperm extraction (micro-TESA). **A** Schematic illustration of the HR reporter system’s working mechanism. **B** Fluorescence-Activated Cell Sorting (FACS) analysis of HR reporter in *MCM9* WT and KO cell lines. **C** Quantification of *Mcm9*^KO^ HEK293T cells with restoration of one active eGFP copy via Sce1-induced HR. **D** Co-immunoprecipitation assays were performed to evaluate the interactions between MCM9 and both MLH1 and MSH2 using human testicular protein extracts. **E** Simplified schematic representing the association between MCM9 deficiency and micro-TESA failure in humans.

To investigate whether impaired HR contributes to abnormal DSB repair caused by MCM9 deficiency, we analysed HR efficiency in WT and *Mcm9*^KO^ HEK293T cells carrying the integrated eGFP-based HR reporter array [28]. In both cell lines, HR events within the genomically integrated HR-EGFP reporter led to the recovery of a functional EGFP copy, which could be induced by Sce1-mediated introduction of site-specific DSBs within the reporter array (Fig. 6A). As expected, *Mcm9*^KO^ cells exhibited a significantly lower HR efficiency than that of WT cells, which is consistent with the findings of DNA repair assays (Fig. 6B, C).

MMR is a critical mechanism that ensures accurate transmission of genetic information by correcting errors introduced during DNA replication and maintaining genomic integrity [29]. Given that MCM9 forms a complex with key components of the MMR pathway, including MSH2, MSH3, and MLH1, to support MMR in human somatic cells [30], we investigated whether MCM9 regulates the MMR pathway in germ cells by interacting with MMR proteins. To this end, we performed MS using human testicular protein extracts. Notably, MS analysis revealed a potential interaction between MCM9, MSH2, and MLH1. This interaction was subsequently confirmed by co-IP assays, which demonstrated that MCM9 co-precipitated with MSH2 and MLH1 from human testicular protein extracts (Fig. 6D). These observations provide compelling evidence that deficiencies in MCM9 compromise HR- and MMR-mediated proofreading mechanisms, impairing DNA damage repair during mammalian spermatogenesis.

### MCM9 deficiency causes sperm retrieval failure in humans following micro-TESE

To investigate whether the *MCM9*-mutant probands exhibited localised spermatogenic foci in their seminiferous tubules, similar to observations in *Mcm9*^KO^ mice [16], micro-TESA was performed to attempt sperm retrieval. However, the mutant testes exhibited only small and atrophied seminiferous tubules (Supplemental Fig. 3) and were devoid of haploid sperm cells, including elongated spermatids and spermatozoa. These results are consistent with the histological diagnosis of SCOS in a patient with NOA and MCM9 deficiency [21], suggesting that MCM9 deficiency leads to a complete SCOS phenotype and unsuccessful sperm retrieval in humans (Fig. 6E).

## Discussion

Defects in MCM9 are potential causative factors for SPGF in humans, with supporting data from mouse model. However, conclusive evidence remains elusive. In this study, we identified two novel homozygous LoF mutations in *MCM9* that is predominantly expressed in human SSCs, spermatogonia, and spermatocytes, in two unrelated patients with NOA, characterised by SCOS. The absence of MCM9 in testicular tissues demonstrates the pathogenicity of these rare mutations. Additionally, the absence of germ cells in mutant seminiferous tubules, coupled with the reduced HR-mediated DNA repair capacity observed in HEK293T cells either lacking MCM9 or overexpressing mutant MCM9 plasmids, as well as potential defects in the MMR pathway in mutant germ cells, highlights the critical role of MCM9 in mammalian spermatogenesis. Specifically, MCM9 is critical for facilitating HR repair and maintaining the MMR pathway, both of which are vital for preserving DNA integrity in germ cells and for supporting their viability and proliferation. Our genetic findings, together with the poor micro-TESE outcomes observed in patients with *MCM9* mutations, provide new perspectives for genetic counselling and clinical therapeutic strategies for NOA.

Cellular DNA is continuously exposed to a diverse range of genotoxic agents, resulting in various forms of DNA damage. In somatic cells, exposure to harmful external factors such as radiation and chemotherapeutic agents frequently induces ICL-related DNA damage, leading to the accumulation of DNA DSBs, genomic instability, and elevated cancer risk [31]. In addition, gametogenesis requires programmed induction of numerous DSBs during meiotic prophase I to exchange paternal and maternal genetic material, which is essential for ensuring genetic diversity [32]. Notably, both pathological DSBs in somatic cells and programmed DSBs in germ cells rely on HR to promote physical connections between chromosomal pairs for DNA repair [33]. Furthermore, errors arising during normal mitotic and meiotic DNA processes, such as DNA recombination and replication, can introduce mismatches, ultimately leading to DNA damage and genomic instability. The MMR system, a highly conserved biological pathway comprising MutS (MSH) and MutL (MLH) homologues, plays a critical role in correcting DNA lesions. This multistep process involves the recognition of base mismatches, recruitment of repair proteins, excision of errors, and subsequent repair of the DNA sequence [34].

MCM9 exhibits sequence homology with MCM2-7 proteins that form a stable hexameric complex essential for genome stability [35] and is implicated in multiple DNA-related processes including DNA replication initiation [36], MMR [30], and HR [27]. In chicken, mouse, and human cells, MCM9 forms a complex with MCM8, which is crucial for HR repair by recruiting RAD51 to sites of DNA damage [16, 27, 37]. Additionally, MCM9 directly interacts with MMR components and promotes the recruitment of MLH1 to chromatin, regulating MMR-mediated DNA damage repair [30]. Thus, MCM9 is essential for DNA damage response and cell survival by enabling efficient DNA repair and maintaining genomic stability. Consistently, meiotic HR repair is compromised in *Mcm9*^KO^ mice during gametogenesis, leading to the complete absence of oocytes in the ovaries and progressive germ-cell depletion in the testes [16]. The early proliferation defects in *Mcm9*^KO^ germ cells are likely rooted in the depletion of embryonic germ cells [15], a phenomenon that is plausibly attributable to compromised DNA damage repair. Given that these primordial germ cells are responsible for maintaining the genetic integrity of future generations, they may be more susceptible to DNA damage and exhibit a lower tolerance for repair defects compared to other cell types [38].

Collectively, these findings indicated a potential association between MCM9 defects and gametogenic failure in humans. Consistent with this, pathogenic MCM9 mutations have been frequently reported as causative factors for POI over the last decade. However, this association is rarely observed in SPGF, particularly in the context of NOA [35]. Intriguingly, our study identified two novel homozygous mutations in *MCM9* in two unrelated patients with NOA, both of whom exhibited complete absence of germ cells, a pathological condition known as SCOS. The pathogenicity of these rare mutations was evidenced by a complete deficiency of testicular MCM9 in probands. Specifically, the nonsense mutation 1891C > T introduces a premature stop codon into the transcript, probably triggering nonsense-mediated mRNA decay [39], resulting in the absence of MCM9. The intronic mutation 1151-1 G > A affected *MCM9* pre-mRNA splicing both in vivo and in vitro. This alteration causes the skipping of exon 7, resulting in a truncated and unstable transcript of *MCM9*, which may prompt mRNA degradation and ultimately disrupt the synthesis of MCM9.

Progressive and severe early proliferation defects in germ cells constitute the primary pathogenesis underlying the phenotype of *Mcm9*^KO^ testes, where approximately 5% of the affected seminiferous tubules display relatively normal spermatogenesis, whereas the majority exhibit SCOS [15, 16]. Consistently, murine scRNA-seq datasets of adult testicular cells reveal the specific expression of MCM9 in SSCs and spermatogonia, indicating its importance in maintaining the viability and proliferation of spermatogonia. Consistent with these findings, we observed predominant MCM9 expression in both human SSCs and spermatogonia. These results, coupled with the complete absence of germ cells in both probands, provide further evidence for the conserved role of MCM9 in supporting the viability and proliferation of spermatogonia in mammals. Notably, germ cell depletion resulting from MCM9 deficiency in humans, which is hypothesised to safeguard genomic integrity in spermatogonia, is closely associated with impaired HR repair and deficient MMR pathways. As demonstrated by our in vitro DNA repair assays, cells overexpressing mutant MCM9 exhibited impaired DSBs repair capabilities, while *Mcm9*^KO^ cells displayed a marked reduction in HR repair efficiency, resulting in an overall diminished DSB repair capacity. Furthermore, the confirmed interaction between human testicular MCM9 and both MSH2 and MLH1 in our study provides compelling evidence supporting the involvement of the MCM9-mediated MMR pathway in maintaining genomic integrity and supporting the viability and proliferation of spermatogonia in humans.

MCM9 deficiency causes a more severe germ cell loss in humans than in mice, leading to a complete SCOS phenotype. This phenotypic difference is probably attributable to the heightened role of MCM9 in maintaining genome stability and supporting the proliferation of human SSCs compared to that of mice. However, considering that the probands in this study were already 32 and 35 years old, it is plausible that a few seminiferous tubules with normal spermatogenesis existed during the early stages of reproductive system maturation in patients with MCM9 deficiency, resembling the progressive depletion of germ cells observed in *Mcm9*^KO^ mice [15, 16]. In addition, unsuccessful sperm retrieval was observed following micro-TESE in both probands, indicating that biallelic LoF mutations in *MCM9* can be recommended as effective non-invasive molecular biomarkers in the clinic to predict the outcomes of micro-TESE preoperatively. Building on previous studies investigating molecular biomarkers for predicting the outcomes of micro-TESE in patients with NOA [12, 13], our findings establish the hypothesis that defects in genes associated with meiotic prophase or meiotic division during spermatogenesis could serve as predictive indicators of unfavourable outcomes of micro-TESE. Further research is required to test these hypotheses.

In summary, we have provided a detailed analysis of the association between the SCOS phenotype, failed sperm retrieval, and MCM9 deficiency in humans. This discovery deepens our understanding of the genetic basis of male infertility and highlights the potential of these findings to inform future diagnostic and therapeutic strategies.

## Materials and Methods

### Study participants

A cohort of 30 Chinese patients with idiopathic NOA from consanguineous families was recruited from the Reproductive Medical Center of the First Affiliated Hospital of Anhui Medical University. All participants had normal chromosomal karyotypes (46, XY) and tested negative for Y-chromosome microdeletions. Semen analyses were performed according to the World Health Organization guidelines (5th Edition) [2]. NOA diagnoses were confirmed using serum sex hormone testing, scrotal ultrasound examination, and testicular histology evaluations.

### Ethical approval

This study was approved by the Ethics Committee of the First Affiliated Hospital of Anhui Medical University (Approval No. P2020-12-36), and informed consent was obtained from all participants and their family members. All methods were performed in accordance with the relevant guidelines and regulations.

### Genetic analysis

Whole-exome sequencing (WES) and bioinformatic analyses were conducted as previously described [40]. Briefly, genomic DNA was extracted from peripheral whole blood samples using a QIAamp DNA Blood Mini Kit (Qiagen, Hilden, Germany). The Agilent SureSelectXT Human All Exon Kit (Agilent, San Jose, CA, USA) and the Illumina HiSeq X-TEN platform (Illumina, San Diego, CA, USA) were used for sequence isolation and capture. Standard assembly (Burrows–Wheeler Aligner, https://bio-bwa.sourceforge.net/), calling (Genome Analysis Toolkit, https://gatk.broad institute.org/hc/en-us), and annotation (ANNOVAR, https://annovar.openbioin formatics.org/en/latest/) were performed. Candidate mutations and their respective parental origins were identified using Sanger sequencing.

### Histopathological analysis

Testicular tissues were fixed in MDF solution overnight, embedded in paraffin, and sectioned into 5-µm-thick slices before being mounted on glass slides. For histological analysis, the slides were de-paraffinised in xylene and stained with haematoxylin and eosin. The prepared slides were examined for histopathological changes.

### Plasmid preparation

Full-length MCM9 cDNA (NM_017696) was amplified from human testis cDNA using polymerase chain reaction (PCR). The PCR product and 3XFlag-pcDNA3.1 plasmids were digested with BclI and XbaI enzymes. The MCM9 sequence was ligated into the plasmid to construct the Flag-MCM9-pcDNA3.1 vector. A mutant version of the vector lacking exon 7 of the human MCM9 coding sequence was also created. All constructs were confirmed using sequencing to ensure the integrity and accuracy of insertions.

### Cell culture and transfection

HEK293T cells were cultured at 37 °C in Dulbecco’s modified Eagle’s medium supplemented with 10% foetal bovine serum and penicillin/streptomycin. Cells were imaged using an LSM800 confocal microscope (Carl Zeiss AG).

### Western blot (WB) analysis

Human testicular samples and cells were lysed using RIPA buffer (P0013B, Beyotime) supplemented with protease and phosphatase inhibitors (P1049, Beyotime). Proteins were separated by sodium dodecyl sulphate-polyacrylamide gel electrophoresis and transferred onto polyvinylidene difluoride membranes (ISEQ00010, Merck Millipore). The membranes were blocked with 5% non-fat milk in Tris-buffered saline containing Tween-20 (TBST) at room temperature for 2 h and incubated overnight at 4 °C with primary antibodies, including rabbit polyclonal anti-MCM8, anti-MCM9, anti-RPA2 (all 1:2000 dilution, ab191914, ab235335, ab76420, all from Abcam), anti-RAD51, and anti-beta-tubulin (both 1:2000 dilution, PA5-27195, MA5-16308, both from Thermo Fisher Scientific). After three washes with TBST, membranes were incubated with horseradish peroxidase-conjugated secondary antibodies for 2 h at room temperature. Protein bands were visualised using High-signal ECL Western Blotting Substrate (Tanon, Shanghai, China).

### DNA repair assay

HEK293T cells were transiently transfected with either wild-type (WT) or mutant MCM9 plasmids using Lipofectamine 3000 (Thermo Fisher Scientific), following the manufacturer’s instructions. After 24 h of incubation, the cells were treated with etoposide (ETO, 5 mg/mL, MedChemExpress) in culture medium at 37 °C for 2.5 h to induce DNA double-strand breaks (DSBs). Post treatment, the medium was replaced with fresh culture medium, and the cells were allowed to recover at 37 °C for either 3 or 6 h before harvesting. WB analysis was conducted to detect phosphorylation of the Ser-139 residue of the histone variant H2AX (γH2AX; Cell Signalling Technology, Massachusetts, USA), which is a sensitive indicator of DSBs. This experiment was repeated at least three times. Changes in γH2AX levels were quantified by analysing the grayscale intensities of the WB bands using ImageJ software (National Institutes of Health).

### Immunofluorescence (IF)

Paraffin-embedded tissue sections were de-paraffinised, rehydrated, and subjected to antigen retrieval by heating in a microwave oven with Improved Citrate Antigen Retrieval Solution (P0083, Beyotime) for 10 min. MCM9 WT and knock out (KO) cells were treated with 10 μM cisplatin for 24 h, with DMSO as a control. The cells were fixed in 4% paraformaldehyde and 0.1% Triton X-100 in PBS for 10 min and permeabilised with 0.5% Triton X-100 in PBS for 3 min at room temperature. The slides and cell samples were blocked in 10% FBS in PBST (0.1% Triton X-100 in PBS) at 4 °C for 2 h. The slides were incubated at 4 °C, followed by incubation with primary antibodies against MCM9 (1:200, ab235335, Abcam) or γH2AX (1:200, ab81299, Abcam). Cell samples were incubated overnight with γH2AX (1:200, ab81299, Abcam) or RPA2 (1:200, ab76420, Abcam) primary antibodies. The slides and cell samples were washed three times with PBST and incubated with Alexa Fluor 555 anti-rabbit (1:1000, Life Technologies, A21429) or Alexa Fluor 488 anti-mouse (1:500, A11029, Life Technologies) secondary antibodies for 2 h. Before imaging, slides were incubated with Hoechst 33342 (1:500, 62249; Thermo Fisher Scientific) for 5 min, washed in PBS, and mounted with glycerol. Images were acquired using an LSM800 confocal microscope (Carl Zeiss AG) and cells with more than 20 foci per cell were counted.

### Tyramide signal amplification fluorescence assays

After deparaffinisation, rehydration, and antigen retrieval, tissue slides were blocked with 10% goat serum (C0265, Beyotime) for 30 min at room temperature. Slides were incubated overnight at 4 °C with the following primary antibodies: rabbit polyclonal anti-SOX9 (1:200, AB5535, Merck Millipore), rabbit polyclonal anti-DDX4 (1:200, 8761S, Cell Signalling Technology), and anti-Lin28A (1:200, ab46020, Abcam). Following three PBS washes, each lasting 5 min, HRP-conjugated secondary antibody was applied for 1 h at room temperature. The slides were incubated with tyramide reagent (1:100, B40955, Thermo Fisher Scientific) for 8 min to amplify the signal catalytically via HRP activity. Slides were blocked with 10% goat serum and incubated overnight at 4 °C with rabbit polyclonal anti-MCM9 (1:200, ab235335, Abcam). For detection, the slides were treated with Alexa Fluor™ 488 donkey anti-rabbit IgG (H + L) (A-11008, Thermo Fisher Scientific) at 1:500 dilution for 2 h at room temperature. Imaging was performed using an LSM980 confocal microscope (Carl Zeiss).

### Construction of *MCM9* knockout cell strain

To generate the *Mcm9*^KO^ cell model using Cas9, we constructed a plasmid containing spCas9 and gRNAs targeting the MCM9 exons. The plasmid was constructed using the pST1374-NLS-flag-linker-Cas9 vector (#44758; Addgene), which was linearised using MfeI. Two sgRNA DNA fragments (CCAACAGCCGAATGGTGGTC and GTGATTGCCGGAACGCTGCC) were inserted. This plasmid was transfected into the HEK293T cell line (Enogene), and the transfected cells were selected using 10 μg/mL blasticidin (Thermo Fisher Scientific). Single clones were sorted via flow cytometry (BD Biosciences), and knockout was confirmed by genotyping and sequencing analysis.

### Cell viability assay

Cell viability was assessed using a CCK-8 cell counting kit (Vazyme Biotech, A311-02-AA) following the manufacturer’s protocol. MCM9 WT and KO HEK293T cell lines were seeded in a 96-well plate and treated with cisplatin (Sigma-Aldrich, 1134357) at the indicated concentrations (0 to 8 μM, with 0 μM as the control) for 24 h. Subsequently, 10 μL of CCK-8 reagent was added to each well. Following a 2-h incubation in a cell incubator, the absorbance was measured at 450 nm using a microplate spectrophotometer (Epoch2 BioTek, Vermont, USA). Cell viability was represented as a percentage relative to the control.

### HR reporter system and flow cytometry analysis

The HR reporter system comprises two plasmid vectors: pcDNA3.1( + )-I-Sce1-mCherry and pcDNA3.1(+)-eGFP-based HR Reporter [28]. The I-SceI DNA fragment, eGFP-based HR reporter DNA fragment, and vector backbone pcDNA3.1(+)-neo were synthesised by GenScript (Nanjing, China). The eGFP-based HR reporter was designed as follows. The full-length eGFP gene (1–720 bp) was divided into three segments: Fragment A (1–150 bp), Fragment B (250–720 bp), and Fragment C (77–494 bp). An I-SceI restriction site was introduced between fragments A and B. Fragment C, located downstream of Fragment B, contained the missing sequence (151–249 bp) between fragments A and B and homologous sequences at the N- and C-termini that corresponded to fragments A and B, respectively. When HR occurs, Fragment C serves as a template to repair the break between fragments A and B caused by I-SceI-induced DSBs, ultimately restoring the full eGFP sequence and leading to eGFP protein expression. After synthesis, the eGFP-based HR reporter was inserted into the pcDNA3.1(+)-neo vector backbone via the NheI and XhoI restriction sites. Similarly, I-SceI was synthesised and inserted with mCherry into the pcDNA3.1(+)-neo backbone.

Upon completion of plasmid construction, the plasmids were transfected into WT and MCM9 knockout HEK293T cell lines using Lipofectamine 3000 (Invitrogen). Stable transfectants were selected using 1 mg/ml of G418. After 48 h, mCherry and eGFP expressions were observed under a microscope. The proportion of eGFP-positive cells was analysed using flow cytometry (BD).

### Immunoprecipitation mass spectrometry (IP-MS)

IP-MS analysis was performed as previously described [41]. Human testicular proteins were digested and immunoprecipitated using anti-MCM9 (ab235335, Abcam) and anti-IgG antibodies (30000-0-AP, Proteintech) and cross-linked with protein G magnetic beads. Peptides were separated using a NanoLC Ultimate 3000 with an EasySpray column and analysed on an Orbitrap Fusion Lumos in DDA mode. Raw data were processed using MaxQuant (version 1.6.1.0) for label-free quantitation, and peptides were searched against the human UniProt database. Common contaminants were removed during the analysis. IP was subsequently confirmed using WB with anti-MLH1 (1:2000, G168-728, BD Biosciences) and anti-MSH2 (1:2000, ab52266, Abcam).

### Analysis of huamn and murine testicular single-cell transcriptome datasets

Single-cell RNA sequencing (scRNA-seq) datasets of healthy adult human [9] and mouse testes [42, 43] were retrieved from public repositories. Single-cell RNA sequencing (scRNA-seq) datasets of healthy adult human and mouse testes were retrieved from public repositories. Cells expressing ≥ 200 genes and genes detected in ≥ 3 cells were retained. Quality control criteria were applied to include only high-quality cells, defined as those with 200–6000 detected genes and mitochondrial gene content below 10%. The data were normalized, scaled, and batch-corrected via Harmony (sample-based grouping). Dimensionality reduction was conducted using t-distributed stochastic neighbor embedding, followed by clustering based on a shared nearest neighbor graph. Clusters were annotated using lineage-specific marker genes, and ambiguous or mixed-lineage populations were excluded. The expression profile of *MCM9* was subsequently analyzed across the annotated testicular cell types.

### Statistical analysis

Statistical analyses were performed using GraphPad Prism version 10.0. Data are presented as the mean ± standard deviation (SD). Two-way analysis of variance (ANOVA) was employed for comparisons involving two independent variables, while differences between two groups were assessed using Student’s t-test. All P values were two-sided, and a P value of less than 0.05 was considered statistically significant. All experiments were independently repeated at least three times to ensure reproducibility.

## Supplementary information

Supplementary Tables

Supplementary figure

Original Data

## Data Availability

The datasets generated and/or analyzed during the current study are available from the corresponding author upon reasonable request.
