# Supplementary material for: MCM9 deficiency impairs DNA damage repair during spermatogenesis, leading to Sertoli cell-only syndrome in humans
Source: Cell Death Discov. 2025 Jul 1;11:292. doi: 10.1038/s41420-025-02581-y (PMC12218035; doi:10.1038/s41420-025-02581-y)
Supplement: Supplementary file 1 — Supplementary Tables [file 41420_2025_2581_MOESM1_ESM.docx]

**Supplementary Table 1–5**

**MCM9 deficiency impairs DNA damage repair during spermatogenesis, leading to Sertoli cell-only syndrome in humans**

Xuan Sha, Xin Zhang, Hao Geng, Yuqian Li, Xun Xia, Guotong Li, Rong Hua, Kuokuo Li, Yang Gao, Qunshan Shen, Rui Guo, Yuping Xu, Xiaojin He, Yunxia Cao, Mingxi Liu, Huan Wu

**Supplementary Table 1. Homozygous mutations in NOA-related genes identified in our cohort.**

| Patients | Gene | cDNA mutation | Protein alteration | Exon | Mutation type | Reference |
| --- | --- | --- | --- | --- | --- | --- |
| AN001 | *FBXO43* | c.1747C>T | p.Gln583X | 4 | Nonsense | Wu *et* *al*[^1^](#_ENREF_1) |
| AN002 |  |  |  |  |  |  |
| AN004 | *MSH4* | c.1552C>T | p.Gln518X | 11 | Nonsense | Tang *et* *al*[^2^](#_ENREF_2) |
| AN005 | *HMF1* | c.3490C>T | p.Gln1164X | 31 | Nonsense | Tang *et* *al*[^3^](#_ENREF_3) |
| AN006 | *TEX15* | c.7202T>A | p.Leu2401X | 8 | Nonsense | Unpublished |
| AN009 | *SHOC1* | c.1652A>G | p.Gln551Arg | 11 | Missense | Unpublished |
| AN011 | *TDRD9* | c.179_186del | p.Q61Gfs | 1 | Frameshift | Unpublished |
| AN013 | *MCM9* | c.1151-1G>A | / | / | Splicing | In this study |
| AN014 | *FANCA* | c.3263C>T | p.S1088F |  | Missense | Tang *et* *al*[^4^](#_ENREF_4) |
| AN016 | *CCDC155* | c.590T>C | p.Leu197Pro | 7 | Missense | Wu *et* *al*[^5^](#_ENREF_5) |
| AN017 | *MEIOB* | c.528+3A>C | / | / | Splicing | Zhu *et* *al*[^6^](#_ENREF_6) |
| AN019 | *RXFP2* | c.1376-12A>G | / | / | Splicing | Ruan *et* *al*[^7^](#_ENREF_7) |
| AN020 | *MCM9* | c.1891C>T | p.Gln631X | 11 | Nonsense | In this study |
| AN022 | *STX2* | c.205+2T>G | / | / | Splicing | Unpublished |
| AN025 | *MOV10L1* | c.139G>A | p.G47S | 3 | Missense | Unpublished |
| AN028 | *SPIDR* | c.208_209del | p.C70fs | 4 | Frameshift | Unpublished |
| AN029 | *MEI1* | a whole-gene deletion  p.F16Sfs*  Frameshift | | | | Unpublished |

Abbreviation: NOA, nonobstructive azoospermia.

Reference

1. Wu H, Zhang X, Shen Q, et al. A homozygous loss-of-function mutation in FBXO43 causes human non-obstructive azoospermia. *Clinical genetics.* 2022;101(1):55-64.

2. Tang D, Xu C, Geng H, et al. A novel homozygous mutation in the meiotic gene MSH4 leading to male infertility due to non-obstructive azoospermia. *American journal of translational research.* 2020;12(12):8185-8191.

3. Tang D, Lv M, Gao Y, et al. Novel variants in helicase for meiosis 1 lead to male infertility due to non-obstructive azoospermia. *Reproductive biology and endocrinology : RB&E.* 2021;19(1):129.

4. Tang D, Li K, Geng H, et al. Identification of deleterious variants in patients with male infertility due to idiopathic non-obstructive azoospermia. *Reproductive biology and endocrinology : RB&E.* 2022;20(1):63.

5. Wu H, Zhang X, Hua R, et al. Homozygous missense mutation in CCDC155 disrupts the transmembrane distribution of CCDC155 and SUN1, resulting in non-obstructive azoospermia and premature ovarian insufficiency in humans. *Human genetics.* 2022;141(11):1795-1809.

6. Zhu X, Hu K, Cheng H, et al. Novel MEIOB pathogenic variants including a homozygous non-canonical splicing variant, cause meiotic arrest and human non-obstructive azoospermia. *Clinical genetics.* 2024;105(1):99-105.

7. Ruan L, Gu M, Geng H, et al. Achieving an optimal pregnancy outcome through the combined utilization of micro-TESE and ICSI in cryptorchidism associated with a non-canonical splicing variant in RXFP2. *Journal of assisted reproduction and genetics.* 2024;41(5):1307-1317.

**Supplementary Table 2. Details of candidate hemizygous and biallelic mutations identified in AN013 and AN020**

| Patients | Gene | Transcript | cDNA Alteration | Amino Acid Alteration | Mutation Type | Mutation Zygosity |
| --- | --- | --- | --- | --- | --- | --- |
|  |  |  |  |  |  |  |
| **AN013** | **MCM9** | **NM_017696** | **c.1151-1G>A** | **—** | **Splicing** | **Hom** |
|  | SLAIN1 | NM_001242868 | c.219_220insGG | p.A73fs | Frameshift | Hom |
|  | AGAP3 | NM_001042535 | c.94_95insGGGG | p.C32fs | Frameshift | Hom |
|  | LMBRD1 | NM_018368 | c.T1192C | p.Y398H | Missense | Hom |
|  | GALNT12 | NM_024642 | c.C719T | p.P240L | Missense | Hom |
|  | PRICKLE3 | NM_001307979 | c.T40C | p.S14P | Missense | Hemi |
|  | ABHD17A | NM_001130111 | c.39delC | p.F13fs | Frameshift | Het |
|  |  | NM_001130111 | c.36_37del | p.L12fs | Frameshift | Het |
|  | SLC9B1 | NM_001100874 | c.A1318T | p.K440X | Nonsense | Het |
|  |  | NM_001100874 | c.C1234T | p.R412X | Nonsense | Het |
| **AN020** | **MCM9** | **NM_017696** | **c.C1891T** | **p.Q631X** | **Nonsense** | **Hom** |
|  | ZNF559 | NM_001202406 | c.C1720T | p.R574X | Nonsense | Hom |
|  | LRRN4 | NM_152611 | c.268delC | p.L90X | Nonsense | Hom |
|  | TMPO | NM_003276 | c.G1486T | p.E496X | Nonsense | Hom |
|  | AMOT | NM_001113490 | c.A797T | p.H266L | Missense | Hemi |
|  | GAL3ST3 | NM_033036 | c.C650T | p.P217L | Missense | Hom |
|  | EHBP1L1 | NM_001099409 | c.G3788T | p.G1263V | Missense | Hom |
|  | SLC22A25 | NM_199352 | c.C607T | p.R203C | Missense | Hom |
|  | GPR158 | NM_020752 | c.C1573T | p.R525W | Missense | Hom |
|  | C17orf78 | NM_173625 | c.T628C | p.C210R | Missense | Hom |
|  | MS4A8 | NM_031457 | c.G367A | p.V123M | Missense | Hom |
|  | LRRC45 | NM_144999 | c.C637T | p.P213S | Missense | Hom |
|  | APIP | NM_015957 | c.283dupA | p.S95Kfs | Frameshift | Hom |
|  | HRCT1 | NM_001039792 | c.316_317insA | p.P106Hfs | Frameshift | Hom |
|  | TAS2R50 | NM_176890 | c.28delT | p.S10Qfs | Frameshift | Hom |

**Supplementary Table 3. Bioinformatic analyses of candidate mutations identified in AN013 and AN020**

| Patients | Gene | cDNA Alteration | Amino Acid Alteration | Allele frequency | | |  | Deleterious prediction | | |
| --- | --- | --- | --- | --- | --- | --- | --- | --- | --- | --- |
|  |  |  |  | 1KGP | ExAC | gnomAD | Mutation Taster | SIFT | Polyphen-2 | CADD |
| **AN013** | **MCM9** | **c.1151-1G>A** | **—** | **NA** | **NA** | **0** | **D** | **NA** | **NA** | **28.6** |
|  | SLAIN1 | c.219_220insGG | p.A73fs | NA | 0 | NA | NA | NA | NA | NA |
|  | AGAP3 | c.94_95insGGGG | p.C32fs* | NA | 0 | NA | NA | NA | NA | NA |
|  | LMBRD1 | c.T1192C | p.Y398H | 0.0018 | 0.0084 | 0.0071 | D | D | D | 28.5 |
|  | GALNT12 | c.C719T | p.P240L | 0.0022 | 0.0005 | 0.0073 | D | D | D | 35.0 |
|  | PRICKLE3 | c.T40C | p.S14P | NA | NA | NA | D | D | P | 26.9 |
|  | ABHD17A | c.39delC | p.F13fs | NA | 1.99×10^-5^ | 4.44×10^-6^ | NA | NA | NA | NA |
|  |  | c.36_37del | p.L12fs | NA | 1.99×10^-5^ | 4.44×10^-6^ | NA | NA | NA | NA |
|  | SLC9B1 | c.A1318T | p.K440X | NA | NA | 3.73×10^-5^ | D | NA | NA | 42 |
|  |  | c.C1234T | p.R412X | NA | NA | 0.0002 | D | NA | NA | 37 |
| **AN020** | **MCM9** | **c.C1891T** | **p.Q631X** | **NA** | **NA** | **NA** | **NA** | **NA** | **NA** | **NA** |
|  | ZNF559 | c.C1720T | p.R574X | 0.0028 | 0.0008 | 0.0009 | Poly | NA | NA | 25.4 |
|  | LRRN4 | c.268delC | p.L90X | 0.0089 | 0.0088 | 0.0044 | NA | NA | NA | NA |
|  | TMPO | c.G1486T | p.E496X | NA | NA | NA | D | NA | NA | 39.0 |
|  | AMOT | c.A797T | p.H266L | NA | NA | NA | D | D | P | 25.2 |
|  | GAL3ST3 | c.C650T | p.P217L | 0.0009 | 0.0005 | 0.0005 | D | D | P | 23.0 |
|  | EHBP1L1 | c.G3788T | p.G1263V | NA | NA | 2.675×10^-5^ | D | D | P | 24.2 |
|  | SLC22A25 | c.C607T | p.R203C | NA | 3.309×10^-5^ | 2.038×10^-5^ | Poly | D | P | 24.4 |
|  | GPR158 | c.C1573T | p.R525W | 0.0036 | 0.0010 | 0.0009 | D | D | P | 34.0 |
|  | C17orf78 | c.T628C | p.C210R | 0.0002 | 1.787×10^-5^ | 1.634×10^-5^ | D | NA | P | 25.3 |
|  | MS4A8 | c.G367A | p.V123M | NA | 0.0002 | 0.0002 | Poly | D | P | 24.0 |
|  | LRRC45 | c.C637T | p.P213S | 0.0013 | 0.0007 | 0.0008 | D | T | P | 23.3 |
|  | APIP | c.283dupA | p.S95Kfs* | NA | NA | NA | NA | NA | NA | NA |
|  | HRCT1 | c.316_317insA | p.P106Hfs* | NA | NA | NA | NA | NA | NA | NA |
|  | TAS2R50 | c.28delT | p.S10Qfs* | NA | NA | NA | NA | NA | NA | NA |

Abbreviations: 1KGP, 1000 Genomes Project; ExAC, Exome Aggregation Consortium; gnomAD, Genome Aggregation Database; CADD, Combined Annotation Depletion; NA, not available; D, disease-causing; Poly, Polymorphism; P, probably damaging; T, tolerable.

**Supplementary Table 4. Expression profiles and functional roles of candidate disease-associated genes identified in AN013 and AN020^a^**

| Patients | Gene | Tissue enriched | Function |
| --- | --- | --- | --- |
| **AN013** | MCM9 | Bone marrow, lymphoid tissues, and testis | Involved in DNA damage and repair mechanisms during mammalian spermatogenesis. |
|  | SLAIN1 | Brain | Might be involved in the regulation of cytoplasmic microtubule dynamics, microtubule organization and microtubule elongation |
|  | AGAP3 | Brain | GTPase-activating protein for the ADP ribosylation factor family |
|  | LMBRD1 | Adrenal gland, liver, and thyroid gland | Developmental protein for endocytosis, gastrulation, and host-virus interaction. |
|  | GALNT12 | Epididymis and Intestine | Glycosyltransferase. |
|  | PRICKLE3 | Prostate and Skin | Developmental protein for cilium biogenesis. |
|  | ABHD17A | Brain | Hydrolase. |
|  | SLC9B1 | Testis | Involved in sperm motility and fertility. |
| **AN020** | MCM9 | Bone marrow, lymphoid tissues, and testis | Involved in DNA damage and repair mechanisms during mammalian spermatogenesis. |
|  | ZNF559 | Widely expressed | May be involved in transcriptional regulation. |
|  | LRRN4 | Adipose tissue and lung | May play an important role in hippocampus-dependent long-lasting memory. |
|  | TMPO | Lymphoid tissue | May be involved in the structural organization of the nucleus and in the post-mitotic nuclear assembly. |
|  | AMOT | Epididymis and tongue | This gene belongs to the motin family of angiostatin binding proteins characterized by conserved coiled-coil domains and C-terminal PDZ binding motifs. |
|  | GAL3ST3 | Brain and thyroid gland | Transfers a sulfate to position 3 of non-reducing beta-galactosyl residues in N-glycans and core2-branched O-glycans. |
|  | EHBP1L1 | Skeletal muscle | May act as Rab effector protein and play a role in vesicle trafficking. |
|  | SLC22A25 | Liver | Predicted to enable transmembrane transporter activity. |
|  | GPR158 | Brain and retina | Predicted to enable G protein-coupled receptor activity. |
|  | C17orf78 | Intestine | Predicted to be integral component of membrane. |
|  | MS4A8 | Fallopian tube and intestine | May be involved in signal transduction as a component of a multimeric receptor complex. |
|  | LRRC45 | Ciliated cells and extravillous trophoblasts | Component of the proteinaceous fiber-like linker between two centrioles, required for centrosome cohesion. |
|  | APIP | Widely expressed | Amino-acid biosynthesis. |
|  | HRCT1 | Adipose tissue and breast | Predicted to be integral component of membrane. |
|  | TAS2R50 | Not detected. | G-protein coupled receptor. |

**^a^** The data presented in this table were systematically retrieved from the Human Protein Atlas database (<https://www.proteinatlas.org/>).

**Supplementary Table 5. Clinical characteristics of the POI proband carrying the homozygous c.1151-1G>A mutation in *MCM9.***

| Clinical characteristics | Proband |  |
| --- | --- | --- |
|  | POI (II-3) | Ref values |
| Age (y) | 29 | — |
| Serum sex hormone levels |  |  |
| FSH (mIU/mL) | 50.4 | 1.5–7.5 |
| LH (mIU/mL) | 22.8 | 1.2–12.7 |
| E_2_ (pg/mL) | 31.7 | 30.0–400.0 |
| T (ng/mL) | 1.52 | 0–2.5 |
| PRL (μIU/mL) | 110.2 | 51.0–580.0 |
| Ultrasonography |  |  |
| Right ovary (mL) | undetectable | >3.5 |
| Left ovary (mL) | undetectable | >3.5 |
| Uterus (length×width×thickness) (cm×cm×cm) | 1.8×1.1×1.0 | 7.5×3.5×3.5 |
| Karyotype | 46 XX | — |

Abbreviations: Ref., reference; FSH, follicle stimulating hormone; LH, luteinizing hormone; E2, estradiol; T, testosterone; PRL, prolactin.
