# Supplementary material for: MCM9 deficiency impairs DNA damage repair during spermatogenesis, leading to Sertoli cell-only syndrome in humans
Source: Cell Death Discov. 2025 Jul 1;11:292. doi: 10.1038/s41420-025-02581-y (PMC12218035; doi:10.1038/s41420-025-02581-y)
Supplement: Supplementary file 2 — Supplementary figure [file 41420_2025_2581_MOESM2_ESM.pdf]

Supplementary Figure 1-4

MCM9 deficiency impairs DNA damage repair during spermatogenesis, leading to Sertoli cell-only syndrome in humans

Xuan Sha, Xin Zhang, Hao Geng, Yuqian Li, Xun Xia, Guotong Li, Rong Hua, Kuokuo Li, Yang Gao, Qunshan Shen, Rui Guo, Yuping Xu, Xiaojin He, Yunxia Cao, Mingxi Liu, Huan Wu

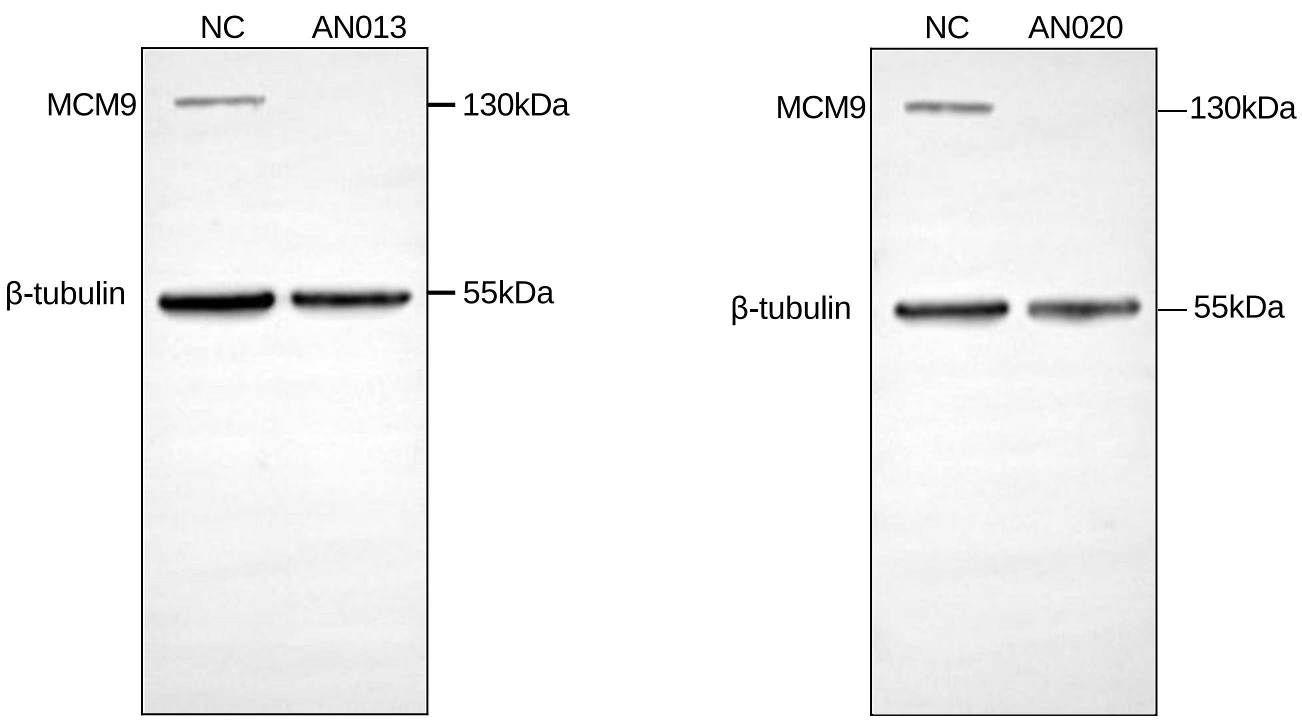

Supplemental Fig. 1. Western blot analysis was performed to evaluate MCM9 expression in testicular samples from two probands and a control male, using β-tubulin as loading control. The results show the complete absence of MCM9 protein in mutant testes, with no detectable truncated forms.

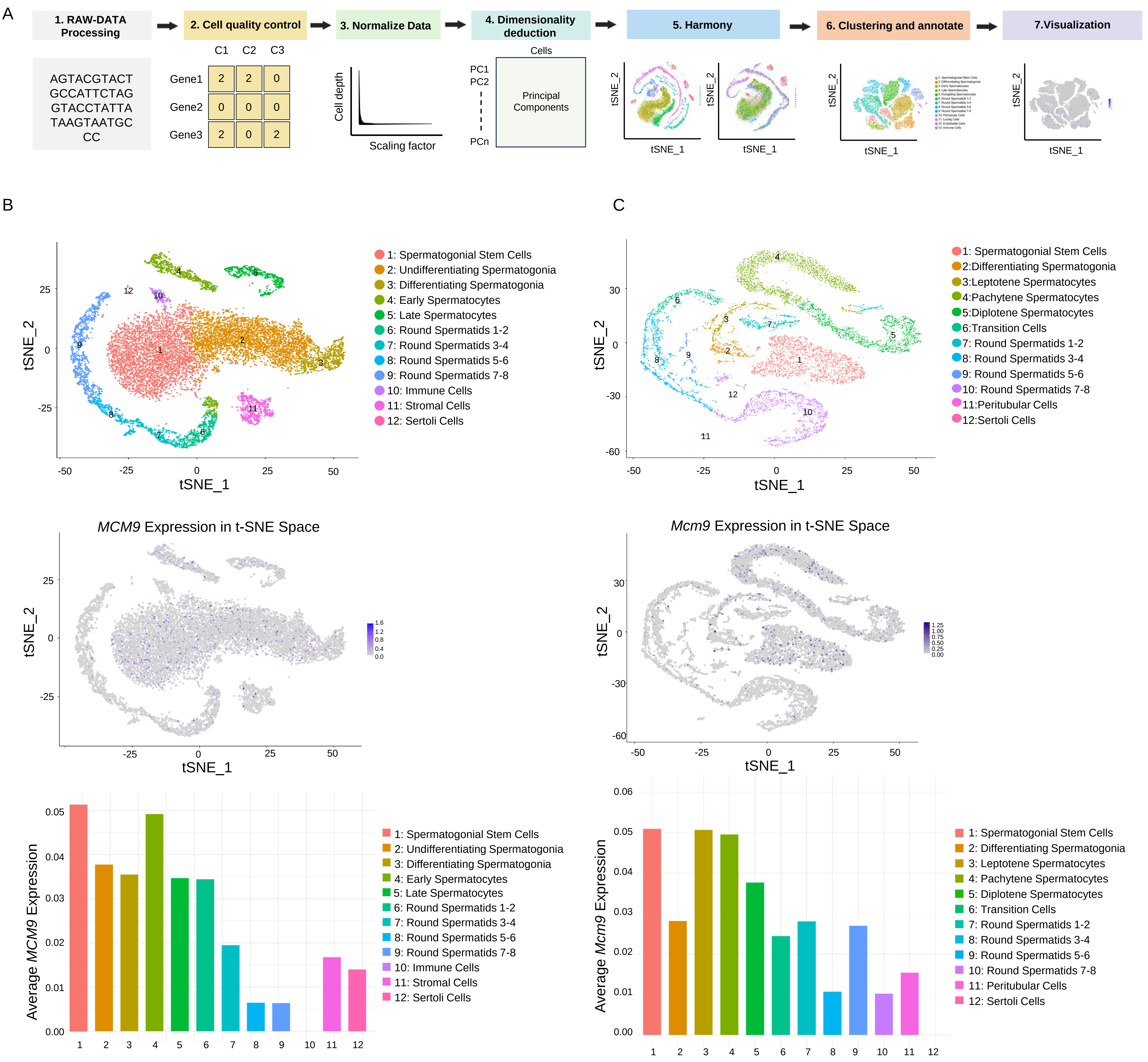

Supplemental Fig. 2. Expression pattern of human and murine MCM9 in testicular cells. (A) Schematic diagram of the single-cell transcriptome dataset analysis workflow. (B-C) Subcellular localization of MCM9 in human and murine testicular cells.

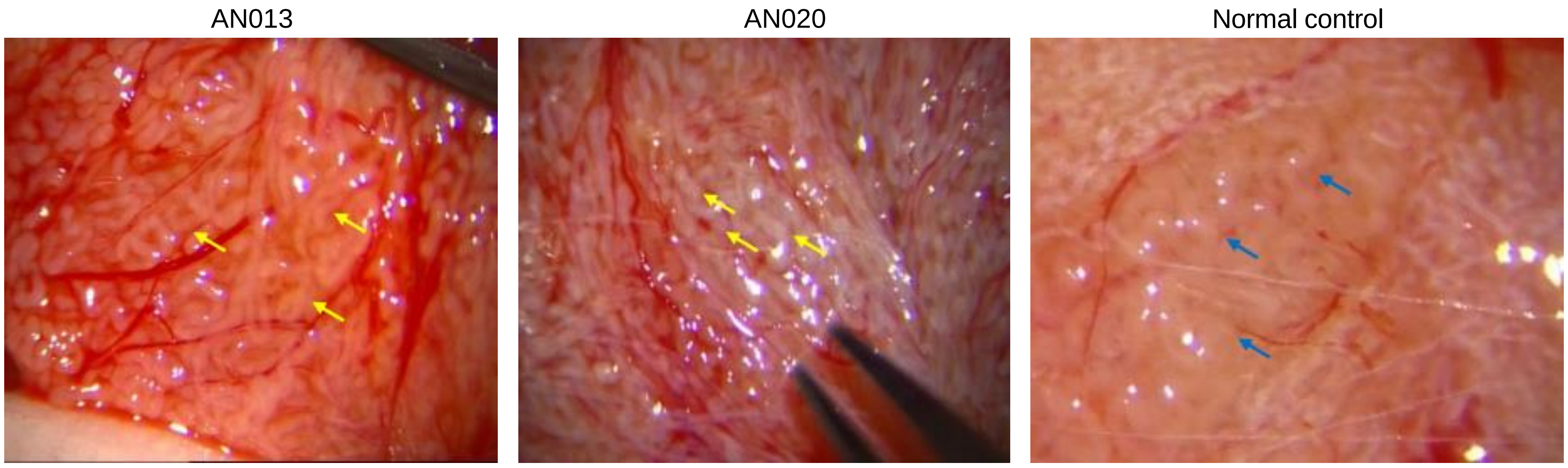

Supplemental Fig. 3. Microdissection testicular sperm extraction (micro-TESE) revealed shrunken seminiferous tubules (yellow arrows) in MCM9-deficient probands, contrasting sharply with the dilated and opaque seminiferous tubules (blue arrows) observed in the control male. Scale bar, 20 μm.

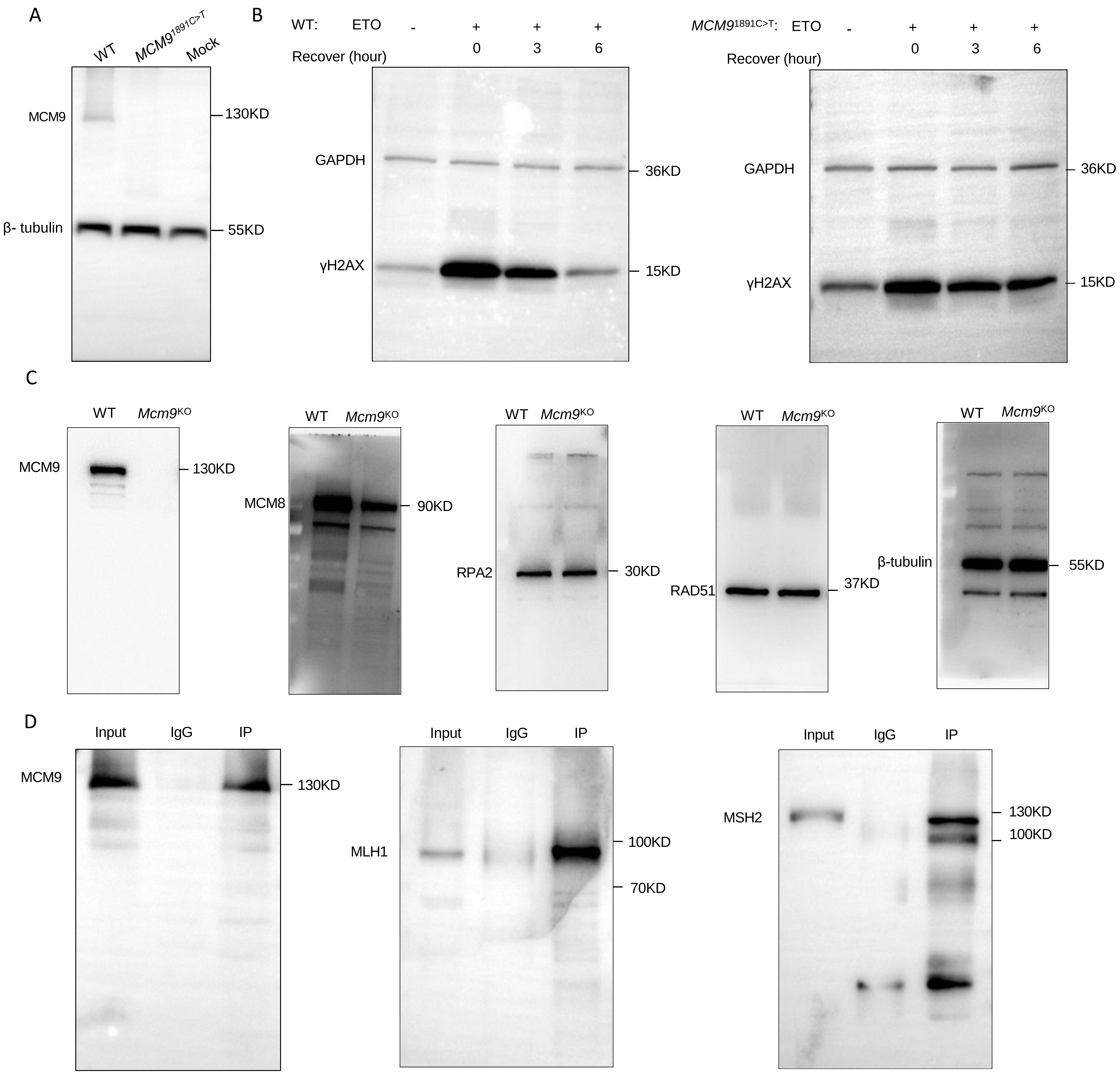

Supplemental Figure 4. The full-length, uncropped original western blot image. A. The full-length, uncropped original western blot image corresponding to Figure 5A.

B. The full-length, uncropped original western blot image corresponding to Figure 5B.

C. The full-length, uncropped original western blot image corresponding to Figure 5E.

D. The full-length, uncropped original western blot image corresponding to Figure 6D
