# Supplementary figures and images for: MCM9 deficiency impairs DNA damage repair during spermatogenesis, leading to Sertoli cell-only syndrome in humans
Source: Cell Death Discov. 2025 Jul 1;11:292. doi: 10.1038/s41420-025-02581-y (PMC12218035; doi:10.1038/s41420-025-02581-y)

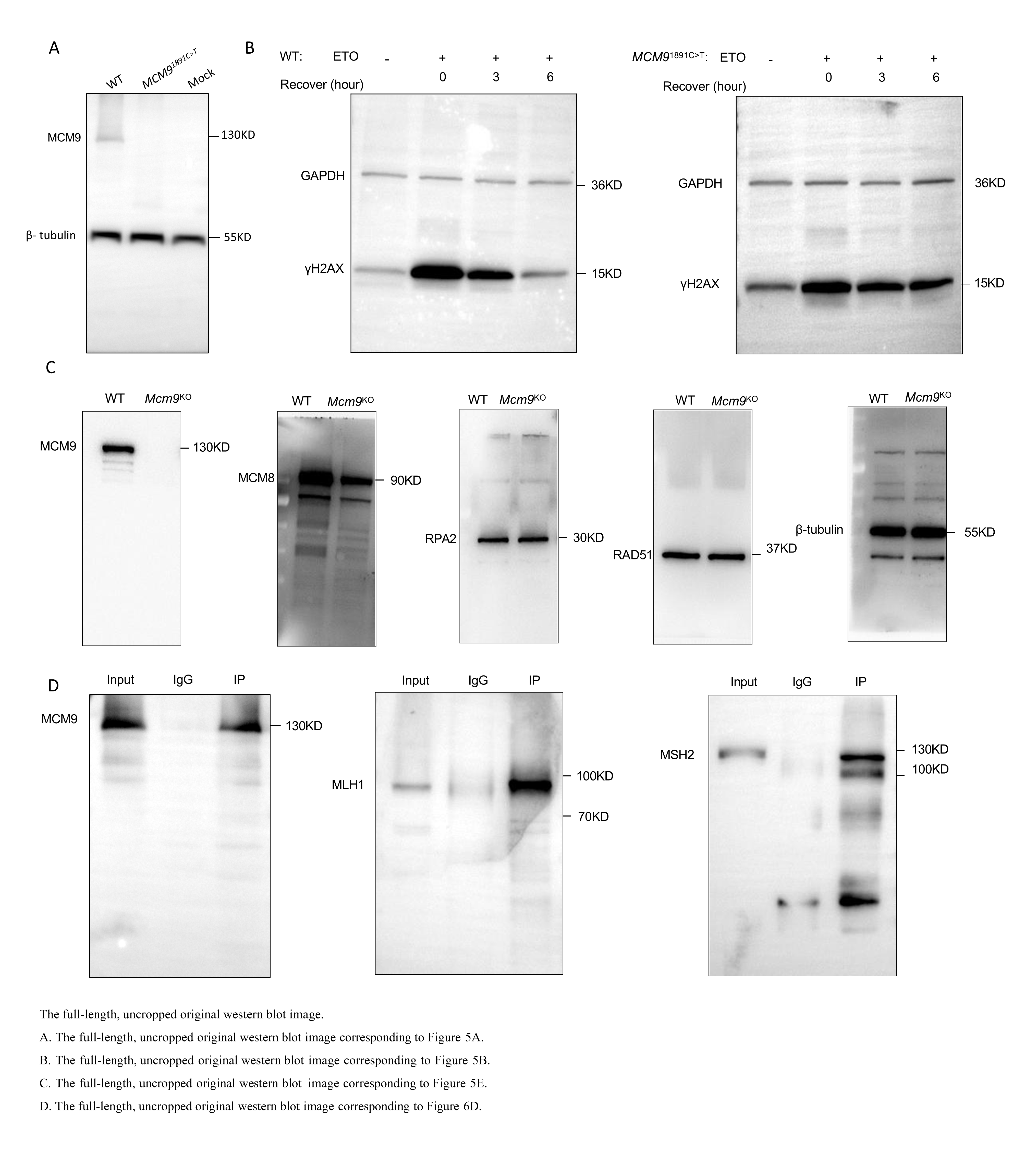

Supplement: Supplementary file 3 — Original Data [file 41420_2025_2581_MOESM3_ESM.tif]
